# Induction and Transcriptome Analysis of Callus Tissue from Endosperm of Makapuno Coconut

**DOI:** 10.3390/plants13223242

**Published:** 2024-11-19

**Authors:** Jing Huang, Zijia Liu, Qinghui Guo, Jixin Zou, Yusheng Zheng, Dongdong Li

**Affiliations:** 1 National Key Laboratory for Tropical Crop Breeding, College of Tropical Agriculture and Forestry, Hainan University, Sanya 572025, China; 18289779930@163.com (J.H.); 183478@hainanu.edu.cn (Z.L.); cmxwgg1@163.com (Q.G.); yusheng.zheng@hainanu.edu.cn (Y.Z.); 2 Rubber Research Institute, Chinese Academy of Tropical Agricultural Sciences (CATAS), Haikou 571101, China; zourich@163.com

**Keywords:** makapuno coconut, endosperm, callus inductin, RNA-seq, lipid

## Abstract

The makapuno coconut endosperm is distinguished by its soft and irregular texture, in contrast to the solid endosperm of regular coconuts. To establish a scientific foundation for studying makapuno coconuts, callus was induced from makapuno endosperm using a combination of plant growth regulators. The induction was successful, and the resulting callus was subsequently subcultured for further study. Transcriptome sequencing of the makapuno callus identified 429 differentially expressed genes (DEGs), with 273 upregulated and 156 downregulated, compared to callus derived from regular coconut endosperm. Kyoto Encyclopedia of Genes and Genomes (KEGG) pathway enrichment analysis indicated that these DEGs were involved in key metabolic pathways, including fructose and mannose metabolism, carbon fixation in photosynthetic organisms, galactose metabolism, and amino sugar and nucleotide sugar metabolism. Furthermore, lipid content analysis of the makapuno callus revealed a significantly higher total lipid level compared to regular callus, with notable differences in the levels of specific fatty acids, such as myristic acid, palmitic acid, and linoleic acid. This study establishes a novel platform for molecular biological research on makapuno coconuts and provides valuable insights into the molecular mechanisms underlying the formation of makapuno callus tissue. The findings also lay the groundwork for future research aimed at elucidating the unique properties of makapuno endosperm and exploring its potential applications.

## 1. Introduction

The coconut (*Cocos nucifera* L.) is a member of the Arecaceae family and the Arecoideae subfamily [1], which includes approximately 27 genera and around 600 species of monocotyledonous plants [2]. The coconut palm serves as a vital economic crop in tropical regions, providing a diverse array of essential products, such as food, beverages, oil, medicine, fiber, and timber [3]. Among its variants, the makapuno coconut represents a unique mutation found in tall-type coconuts [4], distinguished by its markedly different endosperm [5]. The coconut water in makapuno nuts is gelatinous and viscous [6], filling the entire cavity, while the flesh is soft and glutinous, features that have attracted significant interest from farmers [7]. First discovered in the Philippines, the name “makapuno” is derived from a Filipino term meaning “tending to fullness”, aptly describing the gel-like state of its endosperm. Notably, not all coconuts from a makapuno-bearing tree exhibit this gelatinous endosperm [8]. This exceptional variety has also been identified in other tropical regions, including India, Thailand, Indonesia, and Malaysia [9]. However, due to its low yield, makapuno coconuts are priced 10 to 20 times higher than regular coconuts [10].

The distinctive texture of makapuno coconut flesh, in contrast to that of regular tall coconuts, is attributed to a deficiency in enzymes responsible for degrading galactomannan, a complex polysaccharide. This enzymatic deficiency significantly impairs the conversion of galactomannan into its constituent sugars, galactose, fructose, and mannose. Three key enzymes are involved in galactomannan degradation: endo-β-mannanase (EC 3.2.1.78), β-mannosidase (EC 3.2.1.25), and α-galactosidase (EC 3.2.1.22) [11]. In normal coconut endosperm, α-galactosidase activity increases as galactomannan content decreases; however, in makapuno endosperm, α-galactosidase is nearly undetectable, with activity levels up to 8300 times lower than those found in the endosperm of regular coconuts at the same developmental stage [12]. This absence of α-galactosidase is critical [13], as it prevents the adequate breakdown of galactomannan, resulting in the characteristic gelatinous texture of makapuno endosperm [10]. This understanding not only elucidates the biochemical basis for the unique properties of makapuno coconuts but also underscores the challenges associated with cultivating this variant. Nonetheless, there is currently a lack of clear reports on the molecular mechanisms underlying this metabolic difference [10].

Callus formation involves the development of undifferentiated plant tissue from explants, which exhibit totipotency—the ability to regenerate into a complete plant under appropriate conditions [14]. This capability allows researchers to induce callus growth in controlled environments, facilitating the production of desired genetic variants or mutants [15]. Unlike many other tree crops, coconuts pose challenges for vegetative propagation through traditional grafting methods due to their size and structural complexity [16]. However, callus induction presents an alternative approach for the clonal propagation of selected coconut varieties [17], enabling the regeneration of whole plants from desirable genotypes [18]. Current research indicates that callus induction in coconuts has primarily utilized explants from inflorescences, leaves, and embryos [19], with inflorescence explants being preferred due to their lower contamination rates and high dedifferentiation potential.

Recently, there has been increasing interest in coconut endosperm, a tissue that has traditionally received limited attention in callus induction studies [20,21,22]. The potential of coconut endosperm to dedifferentiate into callus tissue remains largely unexplored, presenting a unique research opportunity [23]. The successful induction and regeneration of callus from coconut endosperm could establish a novel platform for studying endosperm biology and enable the clonal propagation of coconuts with specific endosperm traits, such as those found in the makapuno variant [24]. This approach would mark a significant shift from traditional coconut propagation methods, which rely on seed propagation and offer limited scope for genetic manipulation [25].

In this study, we establish a novel research platform centered on the makapuno coconut endosperm by inducing callus formation. This innovative approach yields accessible and versatile material for investigating the biology of makapuno coconut endosperm, thus enabling a wider range of molecular biology experiments. Furthermore, the induced makapuno callus was subjected to transcriptome sequencing to generate comprehensive gene expression data [26]. This integrated methodology aims not only to elucidate the biological mechanisms underlying the distinct traits of makapuno coconuts but also to provide a robust foundation for future research and applications. The establishment of this research platform has the potential to significantly enhance our understanding of makapuno coconuts and facilitate the development of targeted breeding and biotechnological strategies [10].

## 2. Results

### 2.1. Induction of Callus Tissue from Makapuno Coconut Endosperm

**Figure 1:**
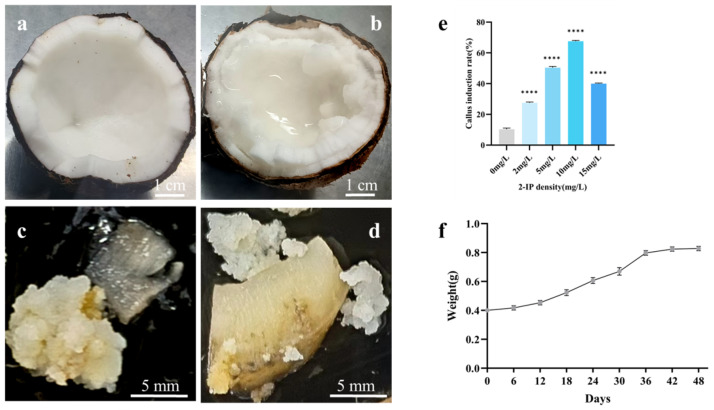
Induction of coconut endosperm callus. (**a**) Reference culture solid coconut endosperm. (**b**) Makapuno solid endosperm. (**c**) Callus induced from reference culture endosperm. (**d**) Callus induced from Makapuno endosperm. (**e**) Effect of 2-IP concentration on callus induction. (**f**) Proliferation of subcultured callus. **** indicates a statistically significant difference (*p* < 0.0001).

To induce callus tissue, endosperm samples were collected from coconuts 9 to 10 months post-pollination. The reference cultivar (RC) endosperm measured approximately 1 cm in thickness, while the makapuno (MK) endosperm was thicker, at around 1.5 cm, indicating a notable morphological difference between the two (Figure 1a,b). The RC endosperm was firm and smooth; whereas, the MK endosperm exhibited a softer and more irregular texture, with a gel-like liquid coating its surface. Callus induction was tested across various concentrations of 2-isopentenyladenine (2-IP), revealing significant differences in induction rates. At concentrations of 0 mg/L, 2 mg/L, and 15 mg/L, callus induction rates were low, with slow growth. In contrast, at 5 mg/L and 10 mg/L, the induction rates increased significantly, reaching 50% and 65%, respectively. While the induction rate initially rose with increasing 2-IP concentrations, it eventually declined at higher levels, indicating that excessive 2-IP inhibited callus growth (Figure 1e). Notably, callus formation was still observed in the absence of 2-IP, but the induction rate was as low as 11%, underscoring that 5 mg/L and 10 mg/L of 2-IP were optimal for effective callus induction.

After six months of incubation, both RC and MK endosperms turned dark brown and developed small, white granular callus tissue on their surfaces (Figure 1c,d). Despite the differences in initial endosperm morphology, no significant phenotypic differences were observed in the callus tissues derived from RC and MK endosperms. This finding suggests that the optimized conditions for callus induction were effective for both types of endosperm, providing a reliable method for generating callus tissue for further studies.

### 2.2. Subculture of Makapuno Callus

The growth dynamics of subcultured makapuno callus were monitored over time by measuring their weight. During the first 36 days, the callus weight showed a positive correlation with the duration of subculture, increasing steadily (Figure 1f). However, from days 36 to 48, no significant changes in callus weight were observed. Over the 48-day period, the weight of an individual callus increased from approximately 0.4 g to 0.8 g, effectively doubling its initial mass. In the first 12 days of subculture, the callus exhibited slow growth, likely due to the adjustment period needed to acclimate to the new culture medium. From days 12 to 36, callus proliferation accelerated significantly, with new white granular callus tissue emerging from the original mass, marking the most rapid phase of growth. However, from days 36 to 48, the growth rate slowed, and callus proliferation gradually plateaued, indicating a cessation of active growth.

These observations suggest that the optimal subculture interval for makapuno callus is between 36 and 42 days post-subculture. At this stage, the callus has completed its proliferation cycle and reached maximum expansion, making it ideal for further subculturing and scaling up. This timing ensures that the callus remains healthy and active, promoting effective propagation and tissue expansion for subsequent experimental applications.

### 2.3. RNA Extraction and RNA-Seq Analysis of Makapuno Callus

**Figure 2:**
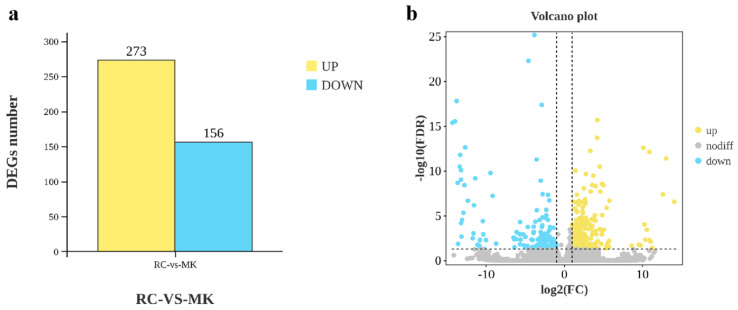
Differentially expressed genes in reference culture and makapuno callus transcriptomes. (**a**) Significantly upregulated and downregulated differentially expressed genes (DEGs) identified in the transcriptomes. (**b**) Gene expression changes between reference culture and Makapuno callus, with upregulated genes shown in yellow and downregulated genes in blue.

RNA was extracted from both reference culture (RC) and makapuno (MK) callus samples, followed by assessments of RNA concentration and purity. Only samples meeting quality standards were utilized for library construction and subsequent RNA-seq analysis. The RNA-seq results revealed a total of 429 significantly differentially expressed genes (DEGs) between RC and MK callus tissues (Figure 2). Among these, 273 DEGs were upregulated, and 156 were downregulated in the MK callus compared to the RC, indicating a predominance of upregulated genes.

**Figure 3:**
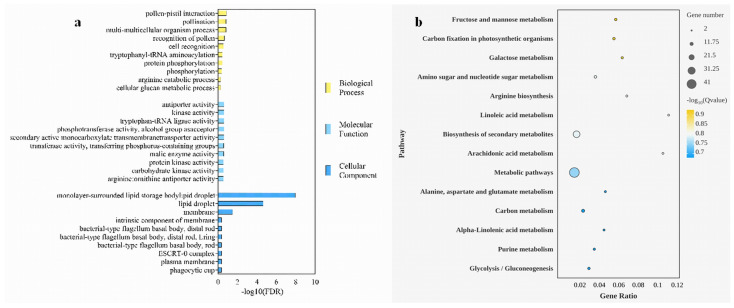
Enrichment analysis of differentially expressed genes in reference culture and makapuno callus. (**a**) Gene Ontology (GO) enrichment analysis of differentially expressed genes in reference culture and makapuno callus. (**b**) Significance bubble plot of KEGG pathway enrichment analysis for reference culture and makapuno callus.

Gene Ontology (GO) enrichment analysis categorized these DEGs into three main categories: molecular function (MF), biological process (BP), and cellular component (CC) (Figure 3a). The BP category contained the highest number of DEGs, with 373 upregulated and 219 downregulated genes, highlighting a significant disparity in the counts of upregulated versus downregulated genes. The MF category included 325 DEGs, with 221 upregulated and 104 downregulated. The CC category had the fewest DEGs, comprising 88 upregulated and 42 downregulated genes (Supplementary Table S1).

GO enrichment analysis indicated significant genetic differences in lipid metabolism between RC and MK callus. Notably, the GO terms “monolayer-surrounded lipid storage body” and “lipid droplet” were highly enriched (*p* < 0.0001), suggesting a pronounced divergence in lipid droplet formation and storage processes between the two callus types. These functional categories were associated with six overlapping DEGs, representing 5.77% of the DEGs enriched in the CC category, indicating a critical role for these genes in the lipid-related physiological differences observed between RC and MK callus. This differential expression pattern provides valuable insights into the molecular mechanisms underlying the unique characteristics of makapuno callus, particularly regarding lipid storage and metabolism. The identification of these DEGs lays a foundation for further investigations into the genetic and metabolic pathways contributing to the distinctive properties of makapuno callus.

### 2.4. KEGG Pathway Analysis of Transcriptome Data

Building on the results of the Gene Ontology (GO) enrichment analysis, a comprehensive KEGG (Kyoto Encyclopedia of Genes and Genomes) pathway analysis was conducted to further explore the functional implications of the differentially expressed genes (DEGs) between reference culture (RC) and makapuno (MK) callus samples. This analysis identified several significantly enriched metabolic pathways (*p* < 0.05) that exhibited notable variations between the two groups, as illustrated in Figure 3b. Among the most significantly enriched pathways were fructose and mannose metabolism (ko00051), carbon fixation in photosynthetic organisms (ko00710), galactose metabolism (ko00052), and amino sugar and nucleotide sugar metabolism (ko00520). The fructose and mannose metabolism pathway included five DEGs, accounting for 7.25% of the annotated pathway genes, while galactose metabolism was represented by four DEGs, constituting 5.8% of the pathway-associated genes. Additionally, two genes were significantly enriched in the linoleic acid metabolism pathway, representing 2.9% of the pathway’s annotated genes.

These findings suggest that the metabolic processes related to carbohydrate and lipid metabolism differ notably between RC and MK callus tissues. Specifically, the alterations in fructose and mannose metabolism, along with changes in galactose metabolism, indicate a potential shift in carbohydrate utilization and storage in the makapuno callus compared to the RC. The enrichment of genes involved in carbon fixation pathways also points to differences in energy metabolism, which may underlie the unique growth characteristics of makapuno callus. In summary, the KEGG pathway analysis enhances our understanding of the metabolic reprogramming occurring in makapuno callus tissue. These insights not only highlight the key pathways differentiating MK from RC but also establish a foundation for future studies aimed at unraveling the specific molecular mechanisms driving the distinctive properties of makapuno coconut callus.

### 2.5. Validation of Differentially Expressed Genes by qRT-PCR

**Figure 4:**
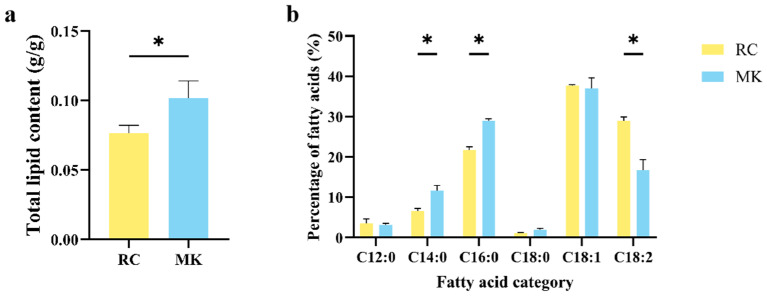
Lipid metabolism analysis in wild-type and makapuno callus. (**a**) Total lipid content per gram of callus in wild-type and makapuno. (**b**) Composition of various fatty acids in the total lipid content of wild-type and makapuno callus. * indicates a statistically significant difference (*p* < 0.05).

To validate the RNA-seq analysis results, several differentially expressed genes (DEGs) associated with key metabolic pathways were selected for quantitative real-time PCR (qRT-PCR) analysis. The selected genes included PFK5 (pfkA, 6-phosphofructokinase 1), PFK3 (pfkA, 6-phosphofructokinase 1), AGAL2 (galA, rafA; alpha-galactosidase), ME6 (maeB; malate dehydrogenase), ALAAT1 (GPT, ALT; alanine transaminase), ARA1 (L-arabinokinase), and OLE1 (At4g25140). The expression levels of these genes were measured using qRT-PCR to confirm the RNA-seq findings. The analysis demonstrated that the expression trends of all selected DEGs were consistent with those observed in the RNA-seq data, validating the reliability of the transcriptomic analysis. In the makapuno (MK) callus, all genes except for ME6 and ARA1 exhibited higher expression levels compared to the reference culture (RC) callus, indicating distinct metabolic activity in the MK callus (Figure 4). Notably, the OLE1 gene, which encodes a protein associated with oil body formation and lipid metabolism, showed significantly higher expression in MK callus than in RC. This result aligns with the higher total lipid content observed in MK callus, suggesting a potential role for OLE1 in regulating lipid accumulation within makapuno callus tissues.

These findings not only confirm the differential expression patterns identified through RNA-seq but also enhance our understanding of the molecular mechanisms underlying the unique metabolic characteristics of makapuno callus. The elevated expression of genes such as OLE1 underscores its potential involvement in lipid biosynthesis and storage, contributing to the distinctive properties of makapuno coconut tissues. Further studies are warranted to explore the functional roles of these key genes in the development and metabolism of makapuno coconut.

### 2.6. Fatty Acid Extraction and Analysis

**Figure 5:**
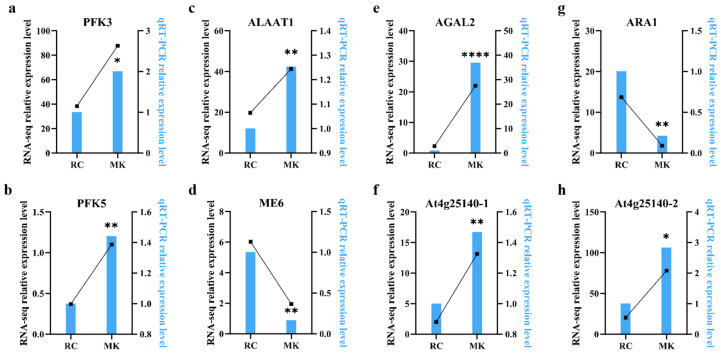
RNA-seq expression and qRT-PCR analysis of candidate genes. (**a**) PFK3 (6-phosphofructokinase 3). (**b**) PFK5 (6-phosphofructokinase 5). (**c**) ALAAT1 (alanine transaminase). (**d**) ME6 (malate dehydrogenase). (**e**) AGAL2 (alpha-galactosidase). (**f**) At4g25140-1 (OLE1). (**g**) ARA1 (L-arabinokinase). (**h**) At4g25140-2 (OLE1). * indicates a statistically significant difference (*p* < 0.05), ** (*p* < 0.01), and **** (*p* < 0.0001).

To further corroborate the differential expression results obtained from the Gene Ontology (GO) and Kyoto Encyclopedia of Genes and Genomes (KEGG) enrichment analyses [27], we conducted a comprehensive evaluation of the fatty acid composition in reference culture (RC) and makapuno (MK) callus tissues. We quantified the total lipid content and the relative percentages of various fatty acids within the total oil content. The results indicated that the total lipid content in the MK callus was significantly higher than that in the RC callus, with MK callus containing approximately 0.10 g of total lipids per gram of tissue, compared to only 0.078 g per gram in RC callus (Figure 5a). Following methylation, the relative abundance of individual fatty acids was determined [28]. As illustrated in Figure 5b, the primary fatty acids identified in both RC and MK callus included lauric acid (C12:0), myristic acid (C14:0), palmitic acid (C16:0), stearic acid (C18:0), oleic acid (C18:1), and linoleic acid (C18:2). Notably, myristic acid, palmitic acid, and linoleic acid exhibited significant differences in their relative proportions between the two callus types. In the MK callus, myristic acid accounted for a 5.09% higher proportion compared to the RC callus, while palmitic acid was elevated by 7.29%. Conversely, the proportion of linoleic acid in the MK callus was 13.21% lower than in the RC callus.

These findings suggest that the elevated total lipid content in the MK callus is accompanied by specific alterations in the fatty acid profile, particularly an increase in saturated fatty acids, such as myristic and palmitic acids, and a decrease in the polyunsaturated fatty acid linoleic acid. The distinct fatty acid composition observed in the MK callus aligns with the RNA-seq data, which indicated a significant enrichment of lipid-metabolism-related pathways. The higher expression of genes involved in lipid biosynthesis, particularly those associated with fatty acid elongation and desaturation, may account for the increased levels of myristic and palmitic acids in the MK callus. The reduced proportion of linoleic acid could be linked to the differential regulation of desaturation pathways, potentially mediated by the lower expression of genes involved in polyunsaturated fatty acid synthesis. This detailed lipid profile analysis not only reinforces the transcriptomic findings but also provides a biochemical basis for the unique metabolic characteristics of makapuno callus. These insights lay the groundwork for future studies aimed at understanding the molecular mechanisms underlying lipid biosynthesis and accumulation in makapuno coconut tissues, which could have significant implications for coconut breeding and cultivation.

## 3. Discussion

This study successfully established an in vitro culture system for inducing embryogenic callus from coconut endosperm, providing a foundational platform for molecular biological research on makapuno coconut [10]. Through high-throughput transcriptome sequencing of reference culture (RC) and makapuno (MK) callus tissues, we identified significant differences in gene expression and metabolic pathways between the two, offering valuable insights into the regulatory genes that may underlie the unique traits of makapuno coconut. Previous studies have emphasized the importance of metabolic pathways, such as fructose and mannose metabolism (ko00051) and galactose metabolism (ko00052), in the context of makapuno coconut research. A key enzyme in these pathways is 6-phosphofructokinase (EC:2.7.1.11), which catalyzes the synthesis of β-D-fructose 1,6-bisphosphate, a crucial step in the glycolytic pathway that influences sugar metabolism in plants. Our findings revealed that the expression levels of PFK5 (pfkA, PFK; 6-phosphofructokinase 1) and PFK3 (pfkA, PFK; 6-phosphofructokinase 1) were significantly upregulated in MK callus compared to RC callus, suggesting enhanced glycolytic activity in makapuno tissues. Although gene expression analysis of the acquired callus tissue can reveal specific differences that reflect the genetic and physiological variations between the two coconut varieties, it is important to note that this callus tissue has undergone a dedifferentiation process, during which it lost some specific characteristics of the original tissue. Consequently, gene expression in the callus tissue may differ from that in the original tissue, potentially influencing the outcomes of transcriptomic comparisons. However, in this study, the same culture conditions were used to induce endosperm callus from both RC and MK varieties, allowing certain metabolic traits characteristic of the original genotypes to be retained. Therefore, transcriptomic and metabolic analyses of the callus tissues can, to some extent, reveal specific metabolic differences between endosperm samples. Such differences may be relevant to breeding programs and commercial applications.

Furthermore, α-galactosidase has long been a focus of research due to its role in the phenotypic differences observed in makapuno coconut endosperm. This enzyme directly impacts the synthesis of galactomannan in coconut water, a key determinant of the gel-like texture characteristic of makapuno coconut. The gene AGAL2 (galA, rafA; alpha-galactosidase), which encodes α-galactosidase, was found to be highly upregulated in mature makapuno endosperm, exhibiting expression levels at least 20 times higher than those in regular coconut endosperm. In our study, the expression level of AGAL2 in MK callus was similarly elevated, with an approximate 30-fold increase compared to RC callus. This significant upregulation aligns with previous findings and further supports the involvement of AGAL2 in the formation of the unique makapuno phenotype. The enhanced expression of these genes in MK callus not only corroborates existing research but also expands our understanding of the molecular mechanisms governing makapuno endosperm development.

The observed differences in metabolic pathways between makapuno (MK) and reference culture (RC) callus provide a comprehensive overview of the metabolic reprogramming occurring in makapuno tissues. This reprogramming likely contributes to the altered carbohydrate and lipid profiles that define the unique texture and composition of makapuno coconut [29]. Future research could focus on the functional validation of key genes through gene editing or overexpression studies, potentially elucidating the direct causal relationships between gene expression, metabolic changes, and the distinctive makapuno phenotype. Additionally, exploring the regulatory networks upstream of these metabolic pathways may identify new targets for enhancing or modifying makapuno traits, thereby providing a broader genetic basis for coconut breeding and genetic improvement efforts.

In this study, the high expression of the OLE1 gene may represent a crucial molecular basis for the unique traits observed in makapuno coconut endosperm. OLE1 encodes oleosin [30], a key protein in seed tissues responsible for maintaining oil body structure and regulating lipid accumulation. One of its primary functions is to stabilize oil bodies, preventing excessive lipid breakdown, while modulating lipid accumulation in seeds and other tissues. In Arabidopsis thaliana, At4g25140 (OLE1) has been shown to significantly influence lipid accumulation in seeds by regulating oil body structure and optimizing the utilization of seed storage substances [31,32]. This function is reflected in makapuno callus tissue as well; our research found that OLE1 expression in makapuno callus was significantly higher than in RC callus, correlating with the elevated total lipid content observed in makapuno callus. While we hypothesize that the upregulation of OLE1 is related to increased lipid content, the specific regulatory mechanisms remain unclear and likely involve a complex network impacting lipid biosynthesis, oil body formation, and lipid transport [33].

Further studies could validate the specific pathways involving OLE1 in makapuno callus by employing various methodologies, such as gene knockout, overexpression, and CRISPR-Cas9 gene editing, to investigate the exact role of the OLE1 gene in lipid accumulation. Additionally, integrating data from metabolomics and lipidomics would elucidate how OLE1 regulates the lipid metabolic network at the molecular level. Investigating the interaction partners of OLE1 and its upstream and downstream regulatory factors could further clarify the molecular mechanisms of lipid metabolism in makapuno coconut endosperm. These findings will not only enhance our understanding of OLE1’s role in the developmental processes of makapuno coconut endosperm but also provide novel insights into the formation of its unique characteristics. This knowledge is essential for improving makapuno coconut cultivation and breeding strategies and for enhancing the economic value of coconut products.

## 4. Materials and Methods

### 4.1. Plant Material Selection

Mature coconuts harvested 9 to 10 months post-pollination from two distinct varieties were utilized as experimental materials. The control group consisted of Wenchang green coconuts, designated as reference culture (RC), sourced from the Coconut Research Institute of the Chinese Academy of Tropical Agricultural Sciences in Wenchang, China. The experimental group included makapuno coconuts, designated as MK, collected from the World Tropical Fruit Window in Qionghai, China. These specific sources ensured that both varieties were grown under comparable environmental conditions, facilitating a more accurate comparison of their biological and molecular characteristics.

### 4.2. Callus Induction and Subculture Procedure

Among the phytohormones that induced endosperm healing in coconut, 2-isopentenyladenine (2-IP), a hormone, was selected, and a concentration gradient of 0 mg/L, 2 mg/L, 5 mg/L, 10 mg/L, and 15 mg/L of 2-IP was set to observe whether the effect of the 2-IP hormone could further promote the generation of healing wounds.

In a 1L volumetric flask, an appropriate amount of MS medium powder was added, then, different concentrations of 2-IP plant hormones were added, and finally, 40 g of sucrose, 2 g of phytogel, and 2.5 g of activated charcoal were weighed and added to the flask. The distilled water was fixed to 1L, and the pH value of the medium was adjusted to 5.8. The prepared medium liquid was put into a sterilizing pot at 121 °C and sterilized for 20 min. The sterilized medium solution was dispensed into 1cm petri dishes in the ultra-clean bench and dispensed into 1cm petri dishes for subsequent inoculation experiments.

Callus induction and subculturing were conducted under carefully controlled aseptic conditions. The process began with the removal of the fibrous husk from the coconuts, leaving the inner endocarp (shell) intact. The coconuts were then sprayed with 75% ethanol and transferred into a sterilized laminar flow hood. To further ensure sterility, they were immersed in 75% ethanol and subjected to UV sterilization for 30 min. Once sterilization was complete, the coconuts were carefully opened in the laminar flow hood. The liquid endosperm was collected in a beaker, while the solid endosperm was extracted using a sterile scalpel and spatula and transferred to a sterile Petri dish. The solid endosperm was then cut into small blocks, each measuring approximately 1 to 1.5 cm in length, and placed onto the induction medium (MS) containing 2-IP with different concentrations to initiate callus formation.

The inoculated endosperm blocks were incubated in darkness to promote callus development, with daily observations to monitor tissue growth and record the time required for callus emergence. Once callus tissue formed, it was carefully detached from the original medium using sterile forceps and placed on filter paper to absorb any residual medium. The callus tissue was then transferred to a fresh subculture medium. Following the initial subculture, the callus tissue was weighed and observed every six days to determine the optimal subculture interval. This step ensured the continuous growth and proliferation of the callus tissue under controlled conditions, providing high-quality material for subsequent experiments.

### 4.3. RNA Extraction and Transcriptome Sequencing of Callus Tissue

Total RNA was extracted from the callus tissue using a combination of mechanical grinding and a specialized RNA extraction kit. The callus samples were initially ground into a fine powder using a mortar and pestle, with liquid nitrogen added to prevent RNA degradation during the process. The powdered tissue was then transferred to RNase-free centrifuge tubes. RNA extraction was performed using the FastPure Plant Total RNA Isolation Kit (RC401-01, Vazyme Biotech, Nanjing, China), according to the manufacturer’s instructions. The extracted RNA was stored at −80 °C for preservation until further analysis.

Quality control assessments were conducted on the RNA samples from both reference culture (RC) and makapuno (MK) callus tissues [34]. Only samples that met quality standards were used for library preparation and subsequent RNA sequencing (RNA-seq). RNA-seq libraries were constructed and sequenced using the Illumina Nova6000 platform (Guangzhou, China). Following sequencing, the raw data underwent stringent quality control to ensure accuracy and reliability [27,35,36]. High-quality reads were aligned to the reference genome using HISAT2 (https://daehwankimlab.github.io/hisat2/, accessed on 25 September 2024), followed by transcript reconstruction. Gene expression levels for each sample were quantified using RSEM [37], resulting in a comprehensive transcriptome profile for both RC and MK callus tissues. This RNA-seq analysis provided valuable insights into the gene expression differences between the two callus types, establishing a foundation for further molecular investigations [38,39].

### 4.4. Differential Gene Screening and Validation

The identification and analysis of differentially expressed genes (DEGs) were conducted based on RNA-seq data obtained from the callus tissues. DEGs were defined as genes exhibiting a false discovery rate (FDR) of less than 0.05 and an absolute log₂ fold change (log₂FC) greater than 1. Following the identification of DEGs, further analyses—including intergroup differential analysis, comparative analysis, Gene Ontology (GO) enrichment, and Kyoto Encyclopedia of Genes and Genomes (KEGG) pathway enrichment—were performed to explore their functional implications. GO enrichment analysis categorized the DEGs into three major domains: biological processes (BP), cellular components (CC), and molecular functions (MF) [40], providing a comprehensive overview of the functional roles of the identified genes. KEGG pathway enrichment analysis was utilized to map the DEGs onto specific metabolic and signaling pathways, offering insights into the biological processes influenced by these genes and their collective impact on cellular functions.

To validate the RNA-seq results, total RNA extracted from reference culture (RC) and makapuno (MK) callus tissues was reverse-transcribed into complementary DNA (cDNA). Quantitative real-time PCR (qRT-PCR) was subsequently performed to measure the expression levels of selected DEGs. Primers for qRT-PCR were designed using Primer Premier 5.0 software, and relative gene expression levels were calculated using the 2^−ΔΔCT^ method (see primer list in Table 1). This validation step ensured the reliability of the RNA-seq data and provided additional confirmation of the differential expression patterns observed between the RC and MK callus samples.

### 4.5. Fatty Acid Extraction, Determination, and Analysis

To analyze the fatty acid composition of callus tissue, approximately 1 g of callus was ground in liquid nitrogen to obtain a fine powder. The powdered sample underwent lipid extraction using a suitable solvent. Tubes completed with nitrogen blowing were weighed before methylation to obtain the total lipid content. Following extraction, the lipid solution was methylated to convert fatty acids into fatty acid methyl esters (FAMEs). The FAMEs were then dissolved in n-hexane in preparation for analysis. Fatty acid composition was determined using a Hewlett–Packard Ultra GC instrument equipped with an HP-FFAP capillary column (30 m × 0.25 mm, 0.25 μm film thickness), which facilitates effective separation of FAMEs. Gas chromatography analysis provided peak retention times and areas for each fatty acid. These data were employed to calculate the fatty acid methyl ester content and determine the relative abundance of each fatty acid as a percentage of the total fatty acids, utilizing an internal standard method. The quantitative analysis was referred to GB/T17377-2008 [41] and the relative percentage of each fatty acid was calculated based on the peak area using the area normalization method. This approach ensures accurate quantification and the detailed profiling of fatty acids, enabling comprehensive comparisons of lipid composition across different callus samples.

### 4.6. Data Analysis

To compare differences across groups, independent samples *t*-tests were employed. Graphs illustrating the results were created using GraphPad Prism5 software. The following symbols were used to indicate significance levels: * for *p* < 0.05, ** for *p* < 0.01 (strongly significant), *** for *p* < 0.001 (very strongly significant), and **** for *p* < 0.0001 (extremely significant).

## Figures and Tables

**Table 1:** Primers for gene qRT-PCR analysis.

Primers	Sequence(5′-3′)
Co-Actin-F	ATGGTGAAGGCTGGATTTGCTGG
Co-Actin-R	GCATCCTTTTGGCCCATCCC
PFK3-F	TCTGTCTTCTACCAACCAACCAA
PFK3-R	CAACCATCATCCAAGCTCTTCTG
PFK5-F	GGTACCTTTCACTTTAGGGGGA
PFK5-R	CAGCAACACAAATGACAGCATT
ALAAT1-F	AATATGCCGTCCGTGGAGAGA
ALAAT1-R	CATCAAAGGGATGCGAACCTG
ME6-F	TTGCTTCCTGTTGTCTACACACC
ME6-R	AGAATTTTTCCCCTTTCTTTCA
AGAL2-F	GCTATCCTTCTTCTTCCTGCTCC
AGAL2-R	CATCTCTCTCTTATAAGTCTCGGCC
At4g25140-1-F	GTTATCCTTCTCACCAGCCCG
At4g25140-1-R	CCGCAGACCGAGAGGAATC
ARA-F	TAAGTTAAGTGGAGCAAGGCGA
ARA-R	CAGCAAGAGAATACCAATCAGGA
At4g25140-2	AGCAGCCAACGTCGCACAG
At4g25140-2	GTTAGCCCCGAGAGCAGCAG

## Data Availability

The original contributions presented in the study are included in the article/Supplementary Materials, further inquiries can be directed to the corresponding author.

## Supplementary Materials

The following supporting information can be downloaded at: https://www.mdpi.com/article/10.3390/plants13223242/s1.
