# Supplementary material for: Induction and Transcriptome Analysis of Callus Tissue from Endosperm of Makapuno Coconut
Source: Plants (Basel). 2024 Nov 19;13(22):3242. doi: 10.3390/plants13223242 (PMC11598300; doi:10.3390/plants13223242)
Supplement: Supplementary file 1 [file plants-13-03242-s001.zip › plants-3253775-supplementary.pdf]

DEGs_name	Enzyme	RC-1 fpkm	RC-2 fpkm	RC-3 fpkm	MK-1 fpkm	MK-2 fpkm	MK-3 fpkm
PFK5	6-phosphofructokinase 1	0.436	0.278	0.393	1.346	0.988	0.963
PFK3	6-phosphofructokinase 1	46.914	50.146	17.74	94.94	87.885	80.168
AGAL2	alpha-galactosidase	4.148	1.571	0.918	19.768	38.242	7.924
ME6	malate dehydrogenase	5.122	5.559	7.361	1.904	1.95	1.999
ALAAT1	alanine transaminase	22.157	22.547	14.248	46.11	46.594	31.19
ARA1	L-arabinokinase	2.73	8.113	30.26	1.596	2.435	1.298
At4g25140-1	OLE1	2.821	2.259	0.927	12.719	16.168	10.481
At4g25140-2	OLE1	16.596	7.505	36.6	56.408	99.778	77.722
